# Analysis of frailty status and its influencing factors in maintenance hemodialysis patients based on the health ecological model

**DOI:** 10.1186/s12882-025-04716-w

**Published:** 2025-12-26

**Authors:** Xuemei Guo, Bingjie Yang, Jingwen Zhang, Jingyan Zhang, Xueming Jing, Min Tan

**Affiliations:** 1 Department of Nephrology, Affiliated Hospital of North Sichuan Medical College, No. 1, Maoyuan South Road, Nanchong, Sichuan 637000 China; 2 School of Nursing, North Sichuan Medical College, Nanchong, Sichuan 637000 China

**Keywords:** Frailty, Health ecological model, Hemodialysis patients, Influencing factors, Backpropagation neural network

## Abstract

**Background:**

Frailty is a significant public health concern in maintenance hemodialysis (MHD) patients.Previous studies have predominantly focused on isolated risk factors, lacking a comprehensive framework to address its multifactorial nature. This study aims to systematically investigate the prevalence and determinants of frailty in MHD patients using the Health Ecological Model (HEM), which integrates five ecological tiers—individual traits, behavioral characteristics, interpersonal networks, living and working conditions, and policy environment—and to develop a backpropagation neural network (BPNN) prediction model for early frailty identification.

**Methods:**

From January to June 2025, a cross-sectional study was conducted using convenience sampling. A total of 2,224 adult patients on maintenance hemodialysis for ≥3 months were recruited from 10 centers in northeastern Sichuan, China. Frailty was assessed using the Fried Frailty Phenotype scale. Independent variables based on the five tiers of the HEM were analyzed using binary logistic regression. A BPNN model was employed to identify the primary predictors of frailty. The performance of the BPNN model was validated and compared against Random Forest and eXtreme Gradient Boosting (XGBoost) algorithms.

**Results:**

The prevalence of frailty among MHD patients was 30.08%. Key risk factors included weekly exercise < 150 min, depression,poor self-rated health, ≥ 3 chronic comorbidities, age ≥75 years, higher education level, sleep disorders, and divorced/widowed marital status. Protective factors included social support, and urban employee basic medical insurance. The BPNN model identified weekly exercise < 150 min,depression and poor self-rated health as primary predictors of frailty (AUC = 0.944).

**Conclusion:**

The HEM provides an innovative multidimensional perspective for unraveling the complexity of frailty in hemodialysis patients. The BPNN offers a robust tool for precise risk stratification.

**Supplementary Information:**

The online version contains supplementary material available at 10.1186/s12882-025-04716-w.

##  Background

Chronic kidney disease (CKD) is a growing public health burden worldwide, exacerbated by aging populations and rising rates of diabetes and hypertension [1, 2]. The WHO’s 2025 resolution prioritized kidney disease as a major non-communicable disease [3]. Maintenance hemodialysis (MHD) is the primary renal replacement therapy for end-stage renal disease (ESRD), with over 916,000 patients registered in mainland China by December 2023 (CNRDS: https://www.cnrds.com). CKD-specific factors, such as uremic toxins, inflammation, oxidative stress may trigger frailty.Symptoms associated with hemodialysis, including imbalance syndrome, hypotension, and malnutrition, may further exacerbate frailty.The prevalence of frailty among hemodialysis patients ranges from 29.6% to 81.5% [4, 5]. Frailty significantly increases the risk of rehospitalization, vascular access complications, depression, falls, fractures, and death [6, 7]. Moreover, frailty is a dynamic and reversible process. Timely identification of frailty in MHD patients can prevent or delay its onset and mitigate associated adverse outcomes.

Frailty is a clinical state resulting from the accumulation of health deficits,characterized by diminished physiological reserves or multi-system dysfunction, leading to increased vulnerability and reduced resistance to stressors.It clinically manifests as weakness, exhaustion, reduced activity, and weight loss [8, 9]. The incidence of frailty varies significantly depending on the assessment tool used.The Fried Phenotype(FP) [10] is the most commonly used method. though tools like the FRAIL scale are equally concise. Multidimensional assessments such as the Tilburg Frailty Index (TFI) and Clinical Frailty Scale (CFS) cover broader psychological and social domains [11, 12]. However,their susceptibility to cultural and socioeconomic confounding factors may pose challenges for multicenter studies. In contrast, FP offers a more objective evaluation of physical function, is operationally simple, and facilitates direct data collection [13]. Nevertheless,FP is rarely used to assess frailty in dialysis patients across multiple centers. The prevalence of frailty among MHD patients assessed using FP in the northeastern Sichuan,China, remains unclear.

Frailty is a multifactorial syndrome arising from complex interactions among individual, interpersonal networks, and broader social environments, and should be assessed through multiple approaches. Previous studies on frailty in hemodialysis patients often lacked a robust theoretical framework, resulting in fragmented and inconsistent conclusions [6, 14]. In recent years, the Health Ecological Model (HEM) has emerged as an influential framework in health practice, particularly in chronic disease management [15–17]. Che et al. [18] applied this theory to identify factors associated with frailty among community-dwelling older adults with multiple chronic conditions,demonstrating that the model provides a comprehensive logical framework for analyzing influencing factors. The model views health outcomes as a dynamic interaction across five levels: individual characteristics, behavioral traits, interpersonal networks, living and working conditions, and policy environments [19]. which aligns closely with the multifactorial, interactive nature of factors leading to frailty. To address limitations in prior research, this study comprehensively evaluates frailty-related factors among MHD patients from a health ecology perspective, providing theoretical support for precision care and personalized interventions.

Based on the HEM theoretical framework, we selected multidimensional factors to construct predictive models. While binary logistic regression is effective in identifying independent risk factors, it has limitations in capturing complex nonlinear relationships and higher-order interactions,which are particularly relevant in multifactorial conditions like frailty.To overcome this, we introduced a backpropagation neural network (BPNN)—an artificial neural network renowned for its performance in classification tasks and multidimensional mapping [20]. BPNN has been extensively applied in medical fields for disease diagnosis [21], prognosis prediction [21], and etiological exploration [22]. By integrating HEM with BPNN, we can accurately identify key predictors and optimize clinical risk stratification for frailty in MHD patients.

This study aims to assess the prevalence of frailty among MHD patients and explore its multidimensional influencing factors from a health ecology perspective. By integrating BPNN analysis to identify key predictors, it provides a theoretical basis for developing targeted, multi-level interventions to prevent and manage frailty in this population.

## Methods

### Study design and participant recruitment

**Fig. 1:**
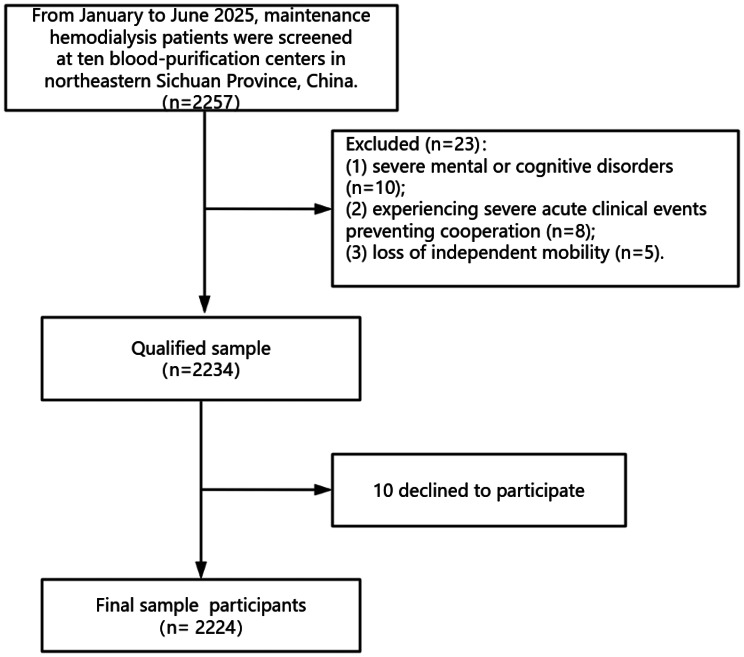
Flowchart for screening research subjects

A cross-sectional survey was conducted from January to June 2025 using convenience sampling across 10 hemodialysis centers (public tertiary, secondary, and private hospitals) in northeastern Sichuan, China. Sampling ensured representation across diverse dialysis regions and hospital types. The screening process is shown in Fig. 1.

**Fig. 2:**
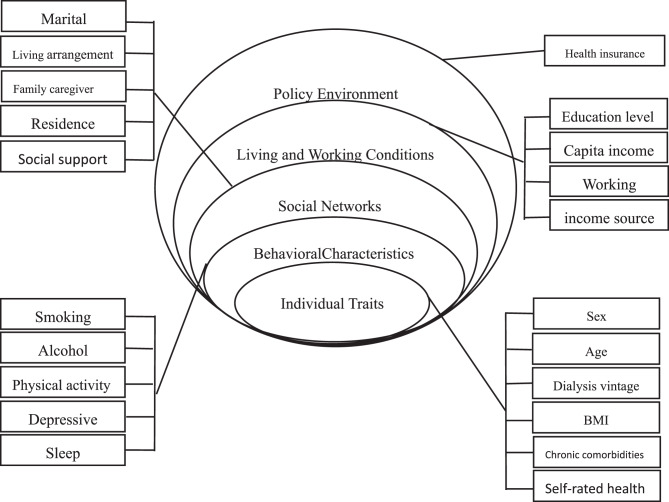
Health ecology model framework

Inclusion Criteria:(1) Age ≥ 18 years;(2) Diagnosed with end-stage renal disease;(3) Undergoing regular hemodialysis for ≥ 3 months;(4) Possessing clear cognitive and communication abilities; (5) Able to complete questionnaires independently or with assistance;(6)Voluntarily participating in the study.

Exclusion Criteria:(1) Individuals unable to move independently; (2) Patients with severe psychiatric disorders; (3) Patients experiencing severe acute clinical events rendering them unable to cooperate.

### Sample size calculation

This study employs a multi-center sampling design, The sample size was calculated using the formula [23]: $n = {{DEFF \times N \times p \times \left( {1 - p} \right)} \over {{d^2}/Z_{1 - \alpha /2}^2 \times \left( {N - 1} \right) + p \times \left( {1 - p} \right)}}$. The parameters were set as follows:N was the number of MHD patients in the CNRDS registry system in mainland China.the design effect (DEFF) was set at 1.5,Z₁₋α/₂ was set at 1.96 (95% significance level, two-tailed test),p was set at 43.3% based on previous prevalence surveys of MHD patients in China [6], and the sampling error (d) was 3%.The recommended sample size was 1,570. Accounting for a potential 20% non-response rate, the sample size was adjusted to 1,963. Ultimately, 2,224 participants were enrolled, exceeding the calculated requirement.

### Measurements

#### Dependent variables

Fried phenotype (FP): Frailty was assessed based on the frailty phenotype criteria by Fried et al. [24], This scale includes five domains:weight loss, slow walking speed, low grip strength, low physical activity and exhaustion. Patients receive 1 point for each criterion met, with a total score ranging from 0 to 5. A score of 0 indicated robust health, 1–2 indicated pre-frailty, and≥3 indicated frailty. The Cronbach’s α coefficient for the scale was 0.821.

Independent Variables (Based on HEM model) (Fig. 2):

1. Individual Traits: Sex, age, dialysis vintage, Body mass index (BMI), chronic comorbidities, self-rated health
2. Behavioral Characteristics: Smoking, alcohol consumption, sleep quality, physical activity, depressive symptoms
3. Interpersonal Networks: Marital status, residence location, ramily caregiver, living arrangement, social support
4. Living and Working Conditions: Education, annual household income per capita, employment status, primary income source
5. Policy Environment: Health insurance

#### Operational definitions

1. Smoking: past or current smoking;
2. Alcohol consumption: past or current drinking;
3. Chronic comorbidities: Presence of ≥ 2 chronic conditions from 13 categories (hypertension,diabetes,dyslipidemia, chronic lung/liver disease, heart disease,stroke, CKD, digestive disorders, mental disorders, memory-related diseases, arthritis/rheumatism, asthma) [25].
4. Physical activity:assessed using the International Physical Activity Questionnaire-Short Scale, and < 150 min/week of exercise defined as inactivity [26].
5. Self-rated health:Categorized as “good” (good/fairly good), “moderate,” or “poor” (poor/fairly poor/very poor).
6. Depression: This study employed the Patient Health Questionnaire-9 (PHQ-9) [27] to measure patients’ discomfort levels over the past two weeks. The scale comprises nine items, each scored from 0 (absolutely none) to 3 (almost every day), with a maximum total score of 27. A total score ≥5 indicates the presence of depressive symptoms, with higher scores reflecting greater severity. The Cronbach’s alpha coefficient for this scale is 0.91; in this study, the Cronbach’s alpha coefficient was 0.831.
7. Sleep disorders:The Insomnia Severity Index (ISI) [27] was employed. This scale comprises seven items, each scored on a 0–4 point scale, yielding a total score range of 0–28 points. A total score of ≥ 8 indicates insomnia, with higher scores reflecting more severe symptoms. The Cronbach’s alpha coefficient for this scale is 0.91; in this study, the Cronbach’s alpha coefficient for this scale was 0.862.
8. Social support:Measured using the Xiao’s [28] Social Support Rating Scale, which comprises three dimensions: objective support (3 items), subjective support (4 items), and support utilization (3 items), totaling 10 items. A total score below 22 indicates low social support, 23–44 indicates moderate social support, and ≥45 indicates high social support. The Cronbach’s alpha coefficient for this scale was 0.861.

#### Quality control

This study employed a questionnaire survey method. Questionnaires were distributed and collected individually on-site through face-to-face interactions. Strict data quality control measures were implemented throughout the collection process.

### Statistical analysis

Statistical analyses were performed using SPSS 26.0 and R software. Categorical variables are expressed as n (%). Intergroup comparisons were conducted using the chi-square test. Continuous variables with normal distribution were expressed as mean ± standard deviation. Binary stepwise logistic regression analyzed factors associated with frailty. Multicollinearity among the independent variables was assessed using variance inflation factors (VIF) and tolerance. A VIF value greater than 5 or a tolerance value less than 0.1 indicates potential multicollinearity concerns.Five nested models were constructed based on the hierarchical health ecology model: Model 1 (Individual characteristics: gender, age, BMI, comorbidities, self-rated health status); Model 2 (Model 1 plus behavioral factors: smoking, alcohol consumption, physical activity, sleep disorders, depression); Model 3 (Model 2 plus interpersonal networks: marital status, place of residence, primary caregiver, living arrangements, social support); Model 4 (Model 3 plus living/working conditions: education level, per capita monthly income, employment status, primary income source); Model 5 (Model 4 plus policy environment: health insurance). In accordance with the hierarchical structure of the HEM and to account for the potential clustering of patients within dialysis centers, multilevel mode was additionally employed. In this model, frailty status was the binary outcome. Patient-level variables from all five tiers of the HEM were included as fixed effects. The dialysis center was included as a random intercept to partition the variance between patient and center levels. The intra-class correlation coefficient (ICC) was calculated to quantify the proportion of total variance attributable to between-center differences.Nested models were compared using Akaike Information Criterion (AIC) and Bayesian Information Criterion (BIC), where lower values indicate better balance between model fit and complexity. Significant predictors (*p* < 0.05) from the final logistic regression model were incorporated as input features into a backpropagation neural network (BPNN), implemented using SPSS’s Multi-layer Perceptron module. comprised an input laye, a hidden layer, and an output layer. Training employed gradient descent with a learning rate of 0.01 and L2 regularization (λ = 0.01) to prevent overfitting. To ensure robust evaluation of model performance, we implemented three complementary validation strategies:First, 10-fold cross-validation was performed for all prediction models. The entire dataset was randomly partitioned into 10 equal-sized folds, with each iteration using 9 folds for training and the remaining fold for validation. Performance metrics are reported as mean ± standard deviation across all 10 folds.Second, to address potential center-specific effects and overfitting concerns in neural networks, we implemented a center-based train-test split. Data from 7 dialysis centers (*n* = 1,558 patients) were used for model training, while data from the remaining 3 centers (*n* = 666 patients) were held out as an independent test set.Third, the predictive performance of the three models was statistically compared using DeLong’s test for paired ROC curves on the center-based test set, with statistical significance defined as *p* < 0.05.

## Results

### Participant characteristics

This study enrolled 2,224 patients with MHD, including 1,333 males (59.94%) and 891 females (40.06%), with an average age of 56.19 years. Among them, 1,287 patients (57.87%) were treated at tertiary hospitals, 606 patients (27.25%) at secondary hospitals, and 331 patients (14.88%) at private hospitals.

### Current status of frailty in MHD patients

**Fig. 3:**
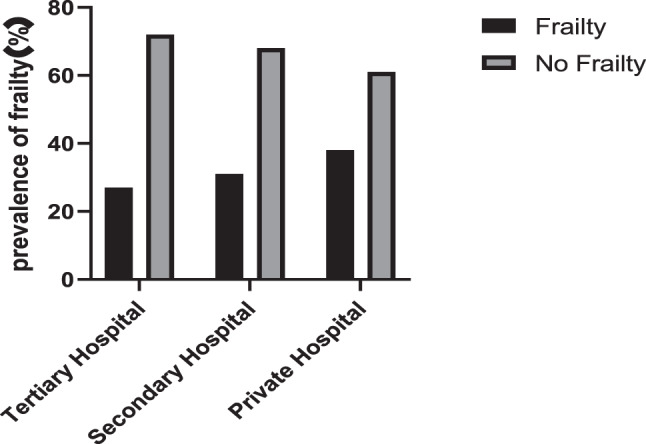
Prevalence of frailty in hospitals of different nature

Among 2,224 MHD patients, 669 exhibited frailty, yielding an overall prevalence rate of 30.08%. Frailty occurred in 127 patients (38.37%) at private hospitals, 188 patients (31.02%) at secondary hospitals, and 354 patients (27.51%) at tertiary hospitals (Fig. 3).

### Group comparison of frailty under different characteristic conditions

Statistically significant differences in frailty were observed across age, chronic disease comorbidity, self-rated health status, physical inactivity, depression, sleep disorders, marital status, place of residence, family caregiver, social support, educational attainment, monthly household income per capita, employment status, and insurance type (*p* < 0.05) (Table 1).

**Table 1:** Frailty detection rate in people with different characteristics(*n* = 2224)

Variable	Number	Frailty	Detection rate（%）	χ^2^	P
Gender				0.000	0.998
Male	1333	401	30.08		
Female	891	268	30.08		
Age（years）				173.439	< 0.001
＜45	413	75	18.16		
45–54	525	106	20.19		
55–64	710	201	28.31		
65–74	406	184	45.32		
≥75	170	103	60.59		
Dialysis Duration (years)				1.339	0.855
≤1	559	177	31.66		
1–3	537	155	28.86		
3–5	367	106	28.88		
5–10	647	197	30.45		
≥10	114	34	29.82		
BMI(kg/m^2^)				0.172	0.982
< 18.5	205	62	30.24		
18.5–23.9	1244	371	29.82		
24.0–27.9	599	184	30.72		
≥28.0	172	51	29.65		
Chronic disease co-morbidity(types)				189.166	< 0.001
1	427	69	16.16		
2	1033	231	22.36		
≥3	764	369	48.30		
Self-rated health				1114.952	< 0.001
Good	151	7	4.64		
Average	1453	152	10.46		
Poor	620	510	82.26		
Smoking				0.129	0.719
Yes	938	286	30.49		
No	1286	383	29.78		
Drinking alcohol				0.009	0.923
Yes	901	270	29.97		
No	1323	399	30.16		
Physical activity				1365.860	< 0.001
Yes	1548	98	6.33		
No	676	571	84.47		
Depression				472.103	< 0.001
Yes	662	414	62.54		
No	1562	255	16.33		
Sleep disorders				205.577	< 0.001
Yes	885	418	47.23		
No	1339	251	18.75		
Marital status				93.964	< 0.001
Unmarried	147	30	20.41		
Married	1823	497	27.26		
Divorced/widowed	254	142	55.91		
Place of residence				27.006	< 0.001
Countryside	814	299	36.73		
City	1410	370	26.24		
Family caregivers				71.417	< 0.001
Self	1244	293	23.55		
Children	149	68	45.64		
Spouse	723	270	37.34		
Parents	76	20	26.32		
Friends or other	32	18	56.25		
Living Arrangements				2.946	0.086
Living alone	338	115	34.02		
Living with others	1886	554	29.37		
Social Support				350.111	< 0.001
Low	421	282	66.98		
Medium	1717	384	22.36		
High	86	3	3.49		
Highest level of education				91.264	< 0.001
Elementary school and below	874	351	40.16		
Junior high school	816	193	23.65		
High School/Middle School	329	53	16.11		
College and above	205	72	35.12		
Monthly household income per capita (CNY)				52.686	< 0.001
< 2500	825	324	39.27		
2500–5000	1301	321	24.67		
＞5000	98	24	24.49		
Working				20.095	< 0.001
Yes	194	31	15.98		
No	2030	638	31.43		
Primary Source of Income				0.128	0.938
Pension or personal savings	720	213	29.58		
Support from Children	1103	334	30.28		
Government Assistance or Other	401	122	30.42		
Medical Insurance				24.630	< 0.001
Urban and rural residents’ Medical insurance	1670	538	32.22		
Urban Employees’ Medical Insurance	515	113	21.94		
Out-of-pocket or Other	39	18	46.15		

### Collinearity diagnosis

Collinearity diagnostics were performed between the frailty status of MHD patients and significant independent variables identified by χ^2^ tests results. The VIF values in the regression analysis were all < 5, indicating no severe multicollinearity among the independent variables.( Supplementary Table 1)

### Logistic regression analysis of factors influencing frailty

The dependent variable was frailty status. Independent variables with significant differences in Table 1 were incorporated into a binary logistic regression models based on the five HEM dimensions. Results showed prediction accuracies were 87.9%, 92.4%, 93.0%, 93.0%, and 92.9% for Models 1–5,Hosmer-Lemeshow test being 13.192, 3.579, 5.284, 4.592, and 8.985, respectively, with significance levels of 0.105, 0.893, 0.727, 0.800, and 0.344. All p-values exceeded 0.05, indicating good model fit. The results of model 5 also showed that age, chronic disease comorbidity, self-rated health,physical activity, depression, sleep disorders, marital status, social support, highest educational attainment, and medical insuranceare major factors influencing frailty occurrence in MHD patients (*p* < 0.05).（Table 2）.Model fitting indices including AIC, BIC, Cox&Snell R^2^,sensitivity, specificity, and other metrics are presented in Supplementary Table 2.

**Table 2:** Regression analysis of influencing factors of frailty

	Model 1		Model 2		Model 3		Model 4		Model 5	
Variables	OR (95% CI)	P	OR (95% CI)	P	OR (95% CI)	P	OR (95% CI)	P	OR (95% CI)	P
Personal trait layer										
Age										
＜45	1.00 (Reference)									
45–54	0.83(0.53~ 1.31)	0.431	0.83 (0.48~ 1.43)	0.497	0.74 (0.39 ~ 1.37)	0.334	0.80(0.42 ~ 1.52)	0.491	0.81 (0.43~ 1.55)	0.525
55–64	1.38(0.92~ 2.10)	0.120	1.21 (0.74 ~ 2.01)	0.449	1.16 (0.64 ~ 2.08)	0.626	1.27 (0.69~ 2.35)	0.444	1.31 (0.71~ 2.44)	0.389
65–74	2.39(1.53~ 3.75)	< 0.001	1.92 (1.10 ~ 3.33)	0.021	1.63 (0.85 ~ 3.11)	0.142	1.84 (0.92 ~ 3.68)	0.084	1.99 (0.98 ~ 4.04)	0.056
≥75	3.41(1.93~ 6.03)	< 0.001	3.86(1.92 ~ 7.73)	< 0.001	3.63 (1.58 ~ 8.36)	0.002	4.11 (1.74 ~ 9.73)	0.001	4.90 (2.01 ~ 11.91)	< 0.001
Chronic disease co-morbidity										
1	1.00 (Reference)									
2	1.56 (1.04 ~ 2.34)	0.031	1.32(0.82 ~ 2.11)	0.256	1.42 (0.85 ~ 2.35)	0.179	1.44 (0.87 ~ 2.40)	0.159	1.47 (0.88 ~ 2.46)	0.142
≥3	4.32 (2.85 ~ 6.54)	< 0.001	3.26 (2.02 ~ 5.27)	< 0.001	3.26 (1.94 ~ 5.47)	< 0.001	3.29(1.95 ~ 5.54)	< 0.001	3.37 (1.99 ~ 5.71)	< 0.001
Self-rated health										
Good	1.00 (Reference)									
Average	1.68(0.76 ~ 3.70)	0.202	3.65 (1.45 ~ 9.21)	0.006	3.07(1.11 ~ 8.49)	0.031	3.32 (1.19 ~ 9.27)	0.022	3.42 (1.21 ~ 9.69)	0.021
Poor	66.37 (29.82~ 147.74)	< 0.001	23.28(9.25~ 58.58)	< 0.001	20.11(7.34 ~ 55.12)	< 0.001	21.69(7.79~ 60.39)	< 0.001	22.64(8.01 ~ 63.95)	< 0.001
Behavioral feature layer										
Physical activity										
No			1.00 (Reference)							
Yes			25.75 (17.97~ 36.90)	< 0.001	26.13 (17.59~ 38.81)	< 0.001	25.40 (17.05~ 37.86)	< 0.001	25.42 (16.98 ~ 38.06)	< 0.001
Depression										
No			1.00 (Reference)							
Yes			1.87 (1.23 ~ 2.85)	0.003	1.78 (1.14~ 2.79)	0.012	1.81(1.15 ~ 2.84)	0.010	1.77 (1.13 ~ 2.78)	0.013
Sleep disorders										
No			1.00 (Reference)							
Yes			2.17 (1.44~ 3.27)	< 0.001	1.85 (1.20 ~ 2.86)	0.006	1.83(1.18~ 2.83)	0.007	1.86 (1.20 ~ 2.89)	0.006
Networking Layer										
Marital status										
Unmarried					1.00 (Reference)					
Married					1.61 (0.71 ~ 3.67)	0.257	1.77(0.77 ~ 4.08)	0.182	1.90 (0.82 ~ 4.43)	0.137
Divorced/widowed					2.58 (1.05 ~ 6.36)	0.040	2.79(1.12 ~ 6.95)	0.028	3.05 (1.21 ~ 7.66)	0.018
Place of residence										
Countryside					1.00 (Reference)					
City					0.85(0.60 ~ 1.23)	0.394	0.81(0.56 ~ 1.19)	0.281	0.87 (0.59~ 1.28)	0.479
Family caregivers										
Self					1.00 (Reference)					
Children					1.31(0.64~ 2.68)		1.36(0.66 ~ 2.79)	0.401	1.34 (0.65 ~ 2.74)	0.429
Spouse					1.47(0.97~ 2.23)		1.44(0.95 ~ 2.19)	0.084	1.46(0.96 ~ 2.21)	0.078
Parents					1.56(0.58~ 4.23)		1.58(0.58~ 4.30)	0.371	1.55 (0.57 ~ 4.24)	0.393
Friends or other					0.31(0.08~ 1.22)		0.32(0.08 ~ 1.30)	0.110	0.30 (0.07 ~ 1.20)	0.089
Social Support										
Low					1.00 (Reference)					
Medium					0.15 (0.10 ~ 0.24)	< 0.001	0.15(0.10 ~ 0.24)	< 0.001	0.15 (0.10 ~ 0.24)	< 0.001
High					0.03(0.01 ~ 0.14)	< 0.001	0.03(0.01~ 0.14)	< 0.001	0.02 (0.01 ~ 0.14)	< 0.001
Living and working conditions layer										
Highest level of education										
Elementary school and below							1.00 (Reference)			
Junior high school							1.02(0.67 ~ 1.56)	0.913	1.07 (0.70 ~ 1.63)	0.748
High School/Middle School							1.14(0.62 ~ 2.10)	0.685	1.26 (0.67 ~ 2.37)	0.469
College and above							2.03(0.97 ~ 4.25)	0.060	2.37 (1.10 ~ 5.12)	0.028
Monthly household income per capita (CNY)										
< 2500							1.00 (Reference)			
2500–5000							0.77(0.54 ~ 1.12)	0.169	0.84 (0.58 ~ 1.22)	0.360
＞5000							0.52(0.17 ~ 1.61)	0.259	0.64 (0.20 ~ 2.08)	0.462
Working										
Yes							1.00 (Reference)			
No							1.05(0.49~ 2.23)	0.904	0.91 (0.43 ~ 1.92)	0.796
Policy environment layer										
Medical Insurance										
Urban and rural residents’ Medical insurance									1.00 (Reference)	
Urban Employees’ Medical Insurance									0.56 (0.34 ~ 0.95)	0.030
Out-of-pocket or Other									0.40 (0.10 ~ 1.53)	0.180

#### Results of the multilevel model analysis

The multilevel model revealed an intra-class correlation coefficient (ICC) of 1.14%, indicating that only a minimal proportion (1.14%) of the total variance in frailty was attributable to differences between dialysis centers. The vast majority of the variance existed at the patient level. After accounting for this center-level clustering, the factors significantly associated with frailty remained largely consistent with those identified in the standard logistic regression analysis (Supplementary Table 3).The goodness-of-fit metrics for the multilevel model indicate AIC, BIC, Conditional R^2^, and Marginal R^2^ values of 958.89, 1,084.45, 0.766, and 0.764, respectively. Additional metrics are detailed in Supplementary Table 4.

#### Neural network model

Using the multi-layer perceptron module of the neural network, statistically significant variables from the multiple regression analysis were sequentially input into the neurons. The system automatically determined the number of neurons in the hidden layer, while the output neurons determined whether frailty occurred. The resulting neural network model comprises an input layer (11 neurons), a hidden layer (3 neurons,hyperbolic tangent (Tanh) activation functions), and an output layer (2 neurons, Softmax activation). The ranking of the importance of each influencing factor is shown in Table 3. Insufficient physical activity emerged as the most influential predictor (standardized importance = 100%), followed by depressive symptoms (80.5%), health insurance type (53.0%), self-rated health (52.8%), age (49.9%), and comorbid chronic conditions (40.2%). Collectively, these factors accounted for the majority of the variance in the model’s predictive ability for frailty status.The feature importance is visualized in Supplementary Figure 1.

**Table 3:** Feature importance ranking in the backpropagation neural network model

Predictor Variable	Importance Score	Normalized Importance (%)
Physical activity	19.33	100.0
Depressive	15.57	80.5
Medical insurance	10.24	53.0
Self-rated health	10.20	52.8
Age	9.65	49.9
Comorbid chronic conditions	7.77	40.2
Social support	3.81	19.7
Sleep disorders	3.14	16.3
Marital status	2.19	11.3
Highest level of education	1.61	8.3

Note: Importance scores were derived from the synaptic weights of the BPNN model trained on data from 7 dialysis centers (*n* = 1,558)

#### Performance evaluation using 10-fold cross-validation

**Fig. 4:**
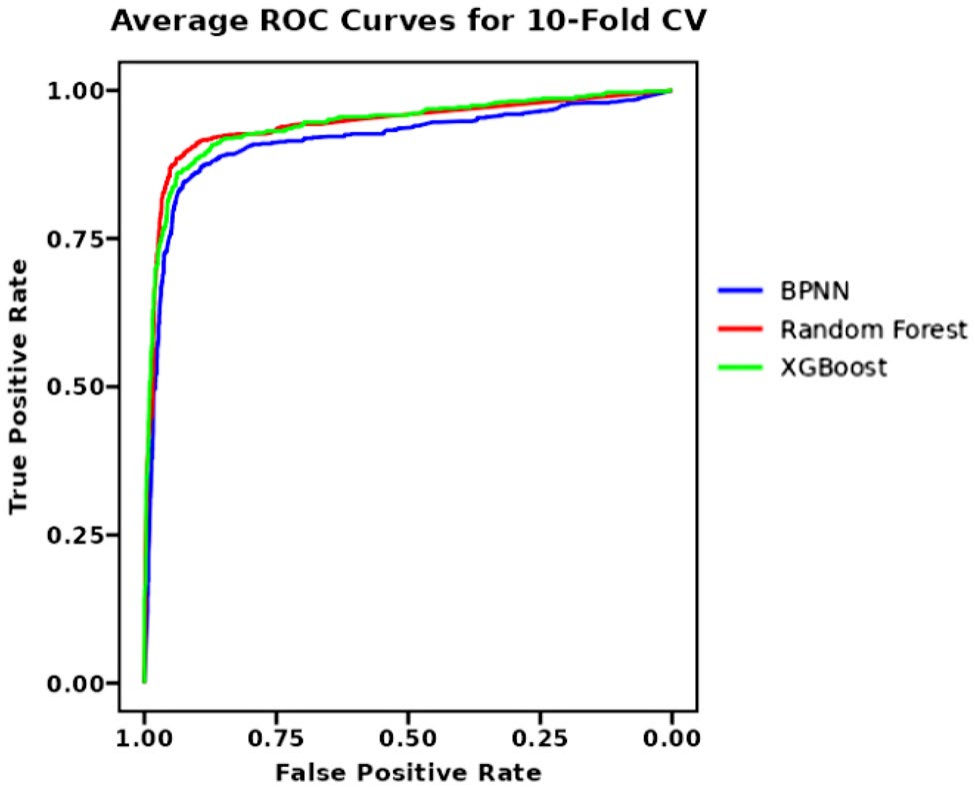
Average ROC curves from 10-fold cross-validation for BPNN, Random forestand XGBoost models

We employ 10-fold cross-validation to provide a robust assessment of the generalization capabilities of BPNN, Random Forest, and XGBoost models.As summarized in Table 4, all three models demonstrated strong and stable predictive performance across the 10 folds.The Random Forest model achieved the highest discriminative ability with a mean AUC of 0.945 (SD = 0.024) and mean accuracy of 0.924 (SD = 0.014), closely followed by XGBoost (mean AUC = 0.943, SD = 0.019; mean accuracy = 0.913, SD = 0.012). Our BPNN model exhibited excellent performance with a mean AUC of 0.918 (SD = 0.027) and mean accuracy of 0.902 (SD = 0.015). The narrow standard deviations indicate consistent performance across different data partitions.The average ROC curve obtained from 10-fold cross-validation is shown in Fig. 4.

**Table 4:** Performance comparison of three prediction models using 10-fold cross-validation

Model	Mean_AUC（±SD）	Mean_Accuracy（±SD）	Mean_Sensitivity（±SD）	Mean_Specificity（±SD）
BPNN	0.918（±0.027）	0.902（±0.015）	0.833（±0.053）	0.933（±0.019）
Random Forest	0.945（±0.024）	0.924（±0.014）	0.851（±0.045）	0.955（±0.011）
XGBoost	0.943（±0.019）	0.913（±0.012）	0.835（±0.046）	0.946（±0.017）

Note: All performance metrics are reported as mean ± standard deviation across 10 cross-validation folds. AUC = Area Under the Receiver Operating Characteristic Curve; SD = Standard Deviation

#### Center-based validation and statistical comparisons

**Fig. 5:**
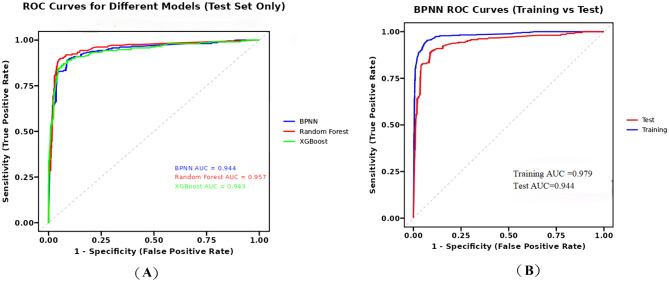
(**a**) Comparison of ROC curves for BPNN, Random Forest, and XGBoost models on the test set; (**b**) ROC curves of the BPNN model on both training and test sets

We adopted a center-based training-testing partitioning approach. The performance of BPNN, Random Forest, and XGBoost models was evaluated on an independent test set comprising three dialysis centers. The BPNN model achieved an AUC of 0.944 and accuracy of 0.910, The Hosmer-Lemeshow test statistic for the training set was 4.189 (*p* = 0.840), with a Brier score of 0.040. For the test set, the Hosmer-Lemeshow statistic was 32.564 (*p* < 0.001), with a Brier score of 0.076. The Random Forest model demonstrated the best performance (AUC = 0.957)and the X-Gated model (AUC = 0.943). All models achieved AUC values exceeding 0.94, indicating they possess outstanding and stable discrimination capabilities.(Fig. 5).Statistical comparison using DeLong’s test insights: there was no significant difference between the BPNN model and either Random Forest (*p* = 0.073) or XGBoost (*p* = 0.941). The only significant difference was between Random Forest and XGBoost (*p* = 0.042). The detailed confusion matrices and calibration plots for the BPNN model on the training and test datasets are presented in Supplementary Table S5 and Supplementary Figure S2, respectively.

## Discussion

In our cross-sectional study, 30.08% of 2224 hemodialysis patients reported frailty.consistent with previous studies reporting frailty prevalence among hemodialysis patients ranging from 13.8% to 41.8% [29]. This rate is higher than the prevalence reported in earlier studies by Ozturk et al. [30], Yuan et al. [31] and Nancy et al. [32], but lower than that reported by Ye et al. [6] and Jiang et al. [33]. The differences may be related to different study areas, different years, different sample sizes, and the use of different frailty assessment tools, as well as the living and dietary habits of different populations.Moreover, frailty is inherently a dynamic process. The BPNN model developed in this study is fundamentally positioned as a cross-sectional screening tool. It aims to efficiently identify the current frailty status of MHD patients based on health ecology theory, utilizing currently available multidimensional indicators.This study indicates that MHD patients with the following characteristics face a higher risk of frailty: weekly exercise duration < 150 min, self-reported poor health status, depression, low social support, age ≥75 years, ≥3 chronic disease comorbidities, sleep disorders, divorce/widowhood, college education or higher, and rural medical insurance.

### Robustness and generalizability of BPNN prediction models

Compared to traditional models commonly used in this field (such as logistic regression), the BPNN algorithm employed in this study is better equipped to capture the complex interactions among factors at different levels within the healthy ecosystem model. This algorithm demonstrated exceptional discriminatory power (AUC = 0.944), and in center-based validation, the BPNN model exhibited statistically equivalent performance to both the Random Forest and XGBoost models (*p* > 0.05). The model performed well in both validation frameworks and captured complex nonlinear relationships, making it a highly competitive and practical solution for frailty screening in MHD patients. However, it is noteworthy that 10-fold cross-validation revealed the BPNN model performed slightly worse than ensemble methods. This indicates that while our BPNN model is competitive, ensemble methods may offer marginally superior stability in certain scenarios. Furthermore, while the BPNN model demonstrated exceptional discriminatory power, its calibration on the independent test set was suboptimal, as evidenced by a significant Hosmer-Lemeshow test (*p* < 0.001). This indicates that predicted probabilities may deviate from actual risk, a common challenge in clinical prediction models. The slightly inferior performance of the BPNN model may stem from its heightened sensitivity to hyperparameter tuning and data features. Nevertheless, all three models achieved AUC values exceeding 0.90, confirming the robust predictive capability of the feature sets constructed based on the theoretical framework.

### Influence of personal traits on frailty in hemodialysis patients

This study indicates that self-reported poor health status, age ≥75 years, and coexisting ≥3 chronic diseases are predictive factors for frailty. We observed that poor self-rated health is a significant factor in frailty development among hemodialysis patients. This may occur because poor self-perceived health often indicates multiple physical discomforts such as fatigue, weakness, and muscle pain. These symptoms can stem from inadequate dialysis, malnutrition, anemia, electrolyte imbalances, and other causes. Prolonged discomfort exacerbates functional impairment, increasing susceptibility to frailty. Patients who perceive their health status as better may also exhibit relatively improved psychological well-being, potentially maintaining more positive attitudes and lifestyles, thereby reducing frailty risk [34]. Furthermore, a clear association exists between advancing age and frailty status, a finding consistent with previous research [35]. Aging, as a major risk factor for frailty, aligns with the cumulative health deficit theory. With increasing age, inevitable decline occurs across bodily systems and organs, leading to reduced physiological reserves and diminished capacity for daily activities. This diminishes stress resilience and increases frailty risk [33]. Furthermore, we observed that having ≥ 3 chronic comorbidities further exacerbates frailty risk, consistent with prior studies [36]. Hemodialysis patients with multiple comorbidities may experience accelerated multi-system functional decline, bear greater comorbidity burdens, and thus hasten progression to frailty.

### Influence of behavioral characteristics on frailty in hemodialysis patients

The results of this study indicate that weekly exercise duration of less than 150 minutes is the most significant risk factor for frailty. Our survey revealed that approximately one-third of patients did not maintain regular exercise. Most patients expressed concerns that exercise might exacerbate their condition, and post-dialysis fatigue and other discomforts may limit their activity levels. Previous studies [37] have found that insufficient physical activity in hemodialysis patients not only leads to reduced peak oxygen uptake but is also associated with muscle weakness and impaired balance. It may further increase anxiety and depression symptoms, foster feelings of social isolation, and elevate the risk of frailty. Therefore, clinical attention should be particularly directed toward slowed walking speed and insufficient physical activity. HD patients exhibiting frailty symptoms urgently require improvements in health status through enhanced exercise capacity, walking speed, and physical activity levels [15].

Research findings further indicate that depression is a significant risk factor for frailty. A Brazilian study revealed that 83.7% of hemodialysis patients exhibited depressive symptoms, with depressed patients facing a 9.8-fold higher risk of frailty compared to non-depressed individuals. Frailty and depression share common pathophysiological underpinnings and influence each other reciprocally. Depression activates inflammatory markers, increasing frailty risk, while frailty itself contributes to the development of depressive symptoms [38].

Furthermore, this study identified sleep disturbances as a risk factor for frailty, consistent with previous research findings [39]. Uremic toxins and inflammatory cytokines can directly affect the brain’s neurotransmitter systems, disrupting normal sleep regulation mechanisms. Poor sleep quality disturbs the hypothalamic-pituitary-adrenal axis, growth hormone secretion, and glucose metabolism. This may further promote muscle catabolism, inhibit muscle synthesis, exacerbate insulin resistance and inflammatory responses, and accelerate the frailty process.Sleep disturbances correlate with alterations in biochemical pathways, including reduced endogenous testosterone levels and elevated inflammatory molecules [40]. These changes may induce protein degradation and muscle proteolysis, thereby increasing frailty risk. Frailty itself can disrupt rest-activity rhythms and irregularize sleep-wake cycles [41]. Consequently, the association between frailty and sleep disturbances is bidirectional, suggesting that assessing sleep disturbances may aid in preventing frailty progression.

### The effect of interpersonal networks on the debilitation of hemodialysis patients

The findings also indicate a significant association between marital status (divorced/widowed) and the risk of frailty, while moderate or higher levels of social support serve as a protective factor against frailty. Consistent with previous studies [42], divorced/widowed patients may have reduced access to various forms of assistance and emotional support, experiencing greater social isolation. This can lead to a series of consequences, such as declining mental health, lack of practical help, reduced quality of life, and increased healthcare burden, which in turn heighten the risk of mental disorders like depression and anxiety, thereby increasing vulnerability to frailty [43]. The buffering effect of social support against chronic disease and dialysis-related stress has been well-documented [44]. Higher levels of social support may help patients cope with disease challenges, alleviate loneliness, reduce the financial strain and psychological burden of dialysis treatment, and enhance psychological resilience against frailty by providing emotional, informational, and material resources [45]. This indicates that social support plays a crucial role in managing frailty among hemodialysis patients. Nurses can collaborate with patients and families to enhance social support and improve the quality of life for dialysis patients.

### Impact of living and working conditions on frailty in hemodialysis patients

The findings of this study also indicate that higher educational attainment is a risk factor for frailty. This conclusion is inconsistent with previous research showing a significant association between frailty and lower educational levels [29]. Such discrepancies may be influenced by variations in environment, population, and geographic location. In this study, patients with higher education levels may have engaged in occupations requiring prolonged sitting. Chronic physical inactivity may accelerate the loss of muscle mass and function, thereby increasing frailty risk [46]. Concurrently, individuals with higher education may harbor greater expectations regarding their health and quality of life. Confronted with the substantial physical decline and life limitations imposed by end-stage renal disease and dialysis treatment, they may experience more severe psychological distress, feelings of loss, or disease-related burdens. These negative emotions themselves constitute significant drivers of frailty [47]. This finding suggests that the role of health-related social determinants among MHD patients may be more complex than in the general population. Future research should incorporate more detailed behavioral psychological indicators and longitudinal data to further elucidate the heterogeneity of education’s impact on health across different populations.

### Impact of the policy environment on debilitation in hemodialysis patients

The findings of this study indicate that, at the policy level, urban employee medical insurance reduces the risk of frailty in MHD patients by 59% (OR = 0.53). This may be attributed to better coverage and access to medical services, as the reimbursement rate under urban employee medical insurance is likely higher than that of basic medical insurance for urban and rural residents. Consequently, patients have greater access to high-quality healthcare, which helps mitigate frailty risk. These results highlight healthcare access as a structural determinant. Future considerations may involve collaborative alliances to achieve cost compensation and improve frailty outcomes for MHD patients [48].

### Limitations

This study has several limitations. First, our assessment of frailty relied on the Fried phenotype scale and did not include biochemical indicators directly linked to the patients’ clinical condition, such as albumin, hemoglobin, or inflammatory markers (e.g., CRP, IL-6).} While the FP is a validated and widely accepted measure that incorporates objective physical performance tests, the inclusion of such laboratory data could provide deeper insights into the biological pathways of frailty and strengthen the clinical relevance of our findings. Our focus on the HEM framework led us to prioritize patient-centered and environmentally oriented measures, which are typically collected via questionnaires and physical tests. Future research would benefit from a combined approach that integrates both comprehensive psychosocial assessments and objective biochemical profiles to fully elucidate the complex etiology of frailty in this population. Secondly, the cross-sectional design of this study did not explore trajectories over time, as longitudinal studies would. Consequently, we cannot establish a causal relationship between risk factors and frailty. Therefore, future research should conduct long-term follow-up studies to investigate the progression of frailty in MHD patients and understand its impact on their prognosis.

## Conclusions

This study employs the Health Ecology Model as its theoretical framework, systematically integrating multidimensional determinants. Utilizing a backpropagation neural network method, it effectively ranks the importance of various factors. Frailty in MHD patients is associated with weekly exercise time < 150 min, self-rated poor health status, depression, low social support, age ≥75 years, ≥3 chronic disease comorbidities, and divorced/widowed marital status, higher education level, lower healthcare policy environment, and sleep disorders. Therefore, while strengthening management of MHD patients, it is crucial to focus on these multidimensional factors. Enhancing exercise capacity, walking speed, and physical activity, improving patients’ subjective health evaluations, and addressing their mental health issues can significantly reduce the risk of frailty and enhance the quality of life for MHD patients.

## Electronic supplementary material

Below is the link to the electronic supplementary material.

Supplementary Material 1

Supplementary Material 2

## Data Availability

The datasets analysed during the current study are available from the corresponding author on reasonable request.
