# Supplementary material for: Analysis of frailty status and its influencing factors in maintenance hemodialysis patients based on the health ecological model
Source: BMC Nephrol. 2025 Dec 26;27:81. doi: 10.1186/s12882-025-04716-w (PMC12849137; doi:10.1186/s12882-025-04716-w)
Supplement: Supplementary file 1 — Supplementary Material 1 [file 12882_2025_4716_MOESM1_ESM.docx]

**Supplementary Material**

Table S1. Collinearity diagnosis Among Independent Variables of Frailty in MHD Patients

|  | **Tolerance** | **VIF** |
| --- | --- | --- |
| Age | **0.726** | **1.377** |
| Chronic disease co-morbidity | **0.927** | **1.079** |
| Self-rated health | **0.837** | **1.195** |
| Physical activity | **0.823** | **1.215** |
| Depression | **0.628** | **1.592** |
| Sleep disorders | **0.627** | **1.594** |
| Marital status | **0.892** | **1.121** |
| Place of residence | **0.869** | **1.150** |
| Family caregivers | **0.889** | **1.125** |
| Social Support | **0.909** | **1.100** |
| Highest level of education | **0.739** | **1.353** |
| Monthly household income per capita | **0.875** | **1.142** |
| Working | **0.853** | **1.172** |
| Medical Insurance | **0.823** | **1.216** |

Table S2. Regression Model Fitting Index

| Model | Deviance | AIC | BIC | df | ΔΧ² | p | McFadden R² | Nagelkerke R² | Tjur R² | Cox & Snell R² | Accuracy | Sensitivity | Specificity |
| --- | --- | --- | --- | --- | --- | --- | --- | --- | --- | --- | --- | --- | --- |
| M₀ | 2,720.2 | 2,722.168 | 2,727.875 | 2,223 |  |  | 0.000 |  | 0.000 |  |  |  |  |
| M₁ | 1,472 | 1,497.554 | 1,571.746 | 2,211 | 1,248.613 | <0.001 | 0.459 | 0.609 | 0.540 | 0.430 | 0.879 | 0.759 | 0.930 |
| M2 | 1,013 | 1,045.241 | 1,136.554 | 2,208 | 1,706.927 | <0.001 | 0.628 | 0.759 | 0.698 | 0.536 | 0.924 | 0.852 | 0.955 |
| M3 | 916.2 | 962.154 | 1,093.416 | 2,201 | 1,804.014 | <0.001 | 0.663 | 0.787 | 0.727 | 0.556 | 0.929 | 0.868 | 0.956 |
| M4 | 910.7 | 968.689 | 1,134.194 | 2,195 | 1,809.479 | <0.001 | 0.665 | 0.789 | 0.728 | 0.557 | 0.931 | 0.870 | 0.958 |
| M5 | 904.6 | 966.645 | 1,143.564 | 2,193 | 1,815.523 | <0.001 | 0.667 | 0.791 | 0.729 | 0.558 | 0.930 | 0.870 | 0.956 |

(Note):M1 (Individual Characteristics Layer: gender, age, BMI, chronic disease comorbidity, self-rated health), M2 (M1 plus Behavioral Characteristics Layer: smoking, alcohol consumption, physical activity, sleep disorders, depression), M3 (M2 plus Network Layer: marital status, place of residence, primary household caregiver, living arrangements, social support), M4 (M3 plus the Living and Working Conditions Layer: educational attainment, monthly household income per capita, employment status, primary source of income), M5 (M4 plus the Policy Environment Layer: health insurance).

| （a）  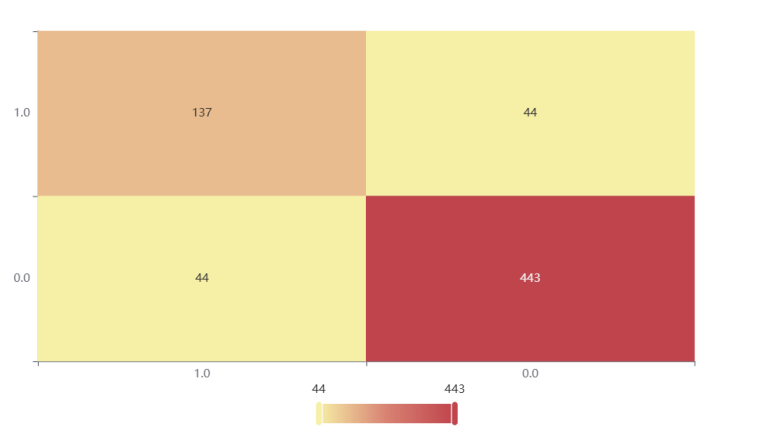  True lable  True lable  Predicted lable | （b）  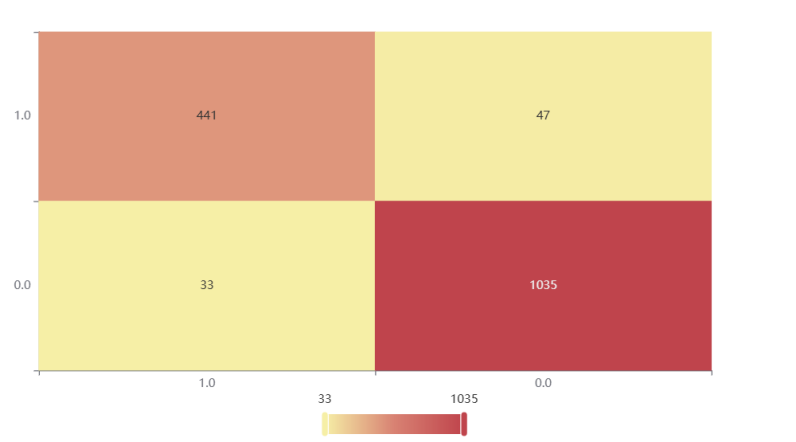  Predicted lable |
| --- | --- |

(Note): Figure S1. Confusion matrices for the BPNN model. (a) Performance on the test set. (b) Performance on the independent training set.

Table S3. Performance Metrics for Training and Test Set Confusion Matrices.

| Metric | Accuracy | Sensitivity (Recall) | Specificity | Precision | F1-Score | AUC |
| --- | --- | --- | --- | --- | --- | --- |
| **Test Set** | 86.8% | 75.7% | 91.0% | 75.7% | 75.7% | 94.2% |
| **Training Set** | 94.9% | 90.3% | 96.9% | 93.0% | 91.6% | 97.8% |

Table S4. Performance Comparison of BPNN with Random Forest and Gradient Boosting Models

| Model | AUC | CA | F1 | Prec | Recall | MCC |
| --- | --- | --- | --- | --- | --- | --- |
| Random Forest | 0.948 | 0.921 | 0.920 | 0.920 | 0.921 | 0.807 |
| Gradient Boosting | 0.962 | 0.928 | 0.928 | 0.928 | 0.928 | 0.825 |
| BPNN | 0.958 | 0.917 | 0.916 | 0.930 | 0.903 | 0.799 |
