# Supplementary material for: Analysis of frailty status and its influencing factors in maintenance hemodialysis patients based on the health ecological model
Source: BMC Nephrol. 2025 Dec 26;27:81. doi: 10.1186/s12882-025-04716-w (PMC12849137; doi:10.1186/s12882-025-04716-w)
Supplement: Supplementary file 2 — Supplementary Material 2 [file 12882_2025_4716_MOESM2_ESM.docx]

**Supplementary Material**

Table S1. Collinearity diagnosis Among Independent Variables of Frailty in MHD Patients

|  | **Tolerance** | **VIF** |
| --- | --- | --- |
| Age | **0.726** | **1.377** |
| Chronic disease co-morbidity | **0.927** | **1.079** |
| Self-rated health | **0.837** | **1.195** |
| Physical activity | **0.823** | **1.215** |
| Depression | **0.628** | **1.592** |
| Sleep disorders | **0.627** | **1.594** |
| Marital status | **0.892** | **1.121** |
| Place of residence | **0.869** | **1.150** |
| Family caregivers | **0.889** | **1.125** |
| Social Support | **0.909** | **1.100** |
| Highest level of education | **0.739** | **1.353** |
| Monthly household income per capita | **0.875** | **1.142** |
| Working | **0.853** | **1.172** |
| Medical Insurance | **0.823** | **1.216** |

Table S2. Regression Model Fitting Index

| Model | Deviance | AIC | BIC | df | ΔΧ² | p | McFadden R² | Nagelkerke R² | Tjur R² | Cox & Snell R² | Accuracy | Sensitivity | Specificity |
| --- | --- | --- | --- | --- | --- | --- | --- | --- | --- | --- | --- | --- | --- |
| M₀ | 2,720.2 | 2,722.168 | 2,727.875 | 2,223 |  |  | 0.000 |  | 0.000 |  |  |  |  |
| M₁ | 1,472 | 1,497.554 | 1,571.746 | 2,211 | 1,248.613 | <0.001 | 0.459 | 0.609 | 0.540 | 0.430 | 0.879 | 0.759 | 0.930 |
| M2 | 1,013 | 1,045.241 | 1,136.554 | 2,208 | 1,706.927 | <0.001 | 0.628 | 0.759 | 0.698 | 0.536 | 0.924 | 0.852 | 0.955 |
| M3 | 916.2 | 962.154 | 1,093.416 | 2,201 | 1,804.014 | <0.001 | 0.663 | 0.787 | 0.727 | 0.556 | 0.929 | 0.868 | 0.956 |
| M4 | 910.7 | 968.689 | 1,134.194 | 2,195 | 1,809.479 | <0.001 | 0.665 | 0.789 | 0.728 | 0.557 | 0.931 | 0.870 | 0.958 |
| M5 | 904.6 | 966.645 | 1,143.564 | 2,193 | 1,815.523 | <0.001 | 0.667 | 0.791 | 0.729 | 0.558 | 0.930 | 0.870 | 0.956 |

(Note):M1 (Individual Characteristics Layer: gender, age, BMI, chronic disease comorbidity, self-rated health), M2 (M1 plus Behavioral Characteristics Layer: smoking, alcohol consumption, physical activity, sleep disorders, depression), M3 (M2 plus Network Layer: marital status, place of residence, primary household caregiver, living arrangements, social support), M4 (M3 plus the Living and Working Conditions Layer: educational attainment, monthly household income per capita, employment status, primary source of income), M5 (M4 plus the Policy Environment Layer: health insurance).

Table S3. Multilevel Model Results for Frailty Predictors

| Characteristic | N | Event N | OR^1^ | 95% CI^1^ | p-value |
| --- | --- | --- | --- | --- | --- |
| **Personal trait layer** |  |  |  |  |  |
| Gender |  |  |  |  |  |
| Male | 1333 | 401 | Reference | - | - |
| Female | 891 | 268 | 0.65 | 0.38, 1.09 | 0.100 |
| Age（years） |  |  |  |  |  |
| ＜45 | 413 | 75 | Reference | - | - |
| 45-54 | 525 | 106 | 0.84 | 0.44, 1.64 | 0.617 |
| 55-64 | 710 | 201 | 1.33 | 0.69, 2.55 | 0.389 |
| 65-74 | 406 | 184 | 1.84 | 0.88, 3.85 | 0.103 |
| ≥75 | 170 | 103 | 4.09 | 1.60, 10.44 | 0.003 |
| Dialysis Duration (years) |  |  |  |  |  |
| ≤1 | 559 | 177 | Reference | - | - |
| 1-3 | 537 | 155 | 1.33 | 0.77, 2.32 | 0.307 |
| 3-5 | 367 | 106 | 1.56 | 0.82, 2.95 | 0.173 |
| 5-10 | 647 | 197 | 1.89 | 1.05, 3.38 | 0.033 |
| ≥10 | 114 | 34 | 0.96 | 0.35, 2.64 | 0.936 |
| BMI(kg/m^2^) |  |  |  |  |  |
| <18.5 | 205 | 62 | Reference | - | - |
| 18.5-23.9 | 1244 | 371 | 0.81 | 0.42, 1.54 | 0.518 |
| 24.0-27.9 | 599 | 184 | 0.78 | 0.38, 1.58 | 0.484 |
| ≥28.0 | 172 | 51 | 0.61 | 0.25, 1.49 | 0.278 |
| Chronic disease co-morbidity(types) |  |  |  |  |  |
| 1 | 427 | 69 | Reference | - | - |
| 2 | 1033 | 231 | 1.45 | 0.84, 2.51 | 0.184 |
| ≥ 3 | 764 | 369 | 3.57 | 2.01, 6.33 | <0.001 |
| Self_rated_health |  |  |  |  |  |
| Poor | 620 | 510 | Reference | - | - |
| Average | 1453 | 152 | 0.15 | 0.10, 0.23 | <0.001 |
| Good | 151 | 7 | 0.05 | 0.02, 0.14 | <0.001 |
| **Behavioral feature layer** |  |  |  |  |  |
| Smoking |  |  |  |  |  |
| No | 1286 | 383 | Reference | - | - |
| Yes | 938 | 286 | 1.01 | 0.52, 1.95 | 0.973 |
| Drinking alcohol |  |  |  |  |  |
| No | 1323 | 399 | Reference | - | - |
| Yes | 901 | 270 | 0.55 | 0.28, 1.05 | 0.070 |
| Physical activity |  |  |  |  |  |
| No | 676 | 571 | Reference | - | - |
| Yes | 1548 | 98 | 0.04 | 0.02, 0.05 | <0.001 |
| Depression |  |  |  |  |  |
| No | 1562 | 255 | Reference | - | - |
| Yes | 662 | 414 | 2.03 | 1.27, 3.27 | 0.003 |
| Sleep disorders |  |  |  |  |  |
| No | 1339 | 251 | Reference | - | - |
| Yes | 885 | 418 | 2.04 | 1.29, 3.24 | 0.002 |
| **Networking Layer** |  |  |  |  |  |
| Marriage |  |  |  |  |  |
| Unmarried | 147 | 30 | Reference | - | - |
| Married | 1823 | 497 | 1.95 | 0.79, 4.82 | 0.149 |
| Divorced/widowed | 254 | 142 | 3.42 | 1.31, 8.98 | 0.012 |
| Place of residence |  |  |  |  |  |
| Countryside | 814 | 299 | Reference | - | - |
| City | 1410 | 370 | 1.16 | 0.76, 1.76 | 0.500 |
| Family caregivers |  |  |  |  |  |
| Self | 1244 | 293 | Reference | - | - |
| Children | 149 | 68 | 1.50 | 0.70, 3.25 | 0.301 |
| Spouse | 723 | 270 | 1.38 | 0.87, 2.20 | 0.173 |
| Parents | 76 | 20 | 1.26 | 0.42, 3.80 | 0.682 |
| Friends or other | 32 | 18 | 0.39 | 0.08, 1.94 | 0.252 |
| Living_Arrangements |  |  |  |  |  |
| Living_alone | 338 | 115 | Reference | - | - |
| Living_with_others | 1886 | 554 | 1.86 | 1.02, 3.39 | 0.044 |
| Social Support |  |  |  |  |  |
| Low | 421 | 282 | Reference | - | - |
| Medium | 1717 | 384 | 0.14 | 0.08, 0.22 | <0.001 |
| High | 86 | 3 | 0.02 | 0.00, 0.15 | <0.001 |
| **Living and working conditions layer** |  |  |  |  |  |
| Highest level of education |  |  |  |  |  |
| Elementary school and below | 874 | 351 | Reference | - | - |
| Junior high school | 816 | 193 | 1.02 | 0.65, 1.58 | 0.937 |
| High School/Middle School | 329 | 53 | 1.17 | 0.61, 2.25 | 0.630 |
| College and above | 205 | 72 | 2.03 | 0.93, 4.39 | 0.074 |
| Monthly household income per capita (CNY) |  |  |  |  |  |
| <2500 | 825 | 324 | Reference | - | - |
| 2500-5000 | 1301 | 321 | 0.70 | 0.46, 1.05 | 0.085 |
| ＞5000 | 98 | 24 | 0.59 | 0.18, 1.93 | 0.379 |
| Working |  |  |  |  |  |
| No | 2030 | 638 | Reference | - | - |
| Yes | 194 | 31 | 1.07 | 0.50, 2.28 | 0.865 |
| Primary Source of Income |  |  |  |  |  |
| Pension or personal savings | 720 | 213 | Reference | - | - |
| Support from Children | 1103 | 334 | 0.53 | 0.31, 0.92 | 0.023 |
| Government Assistance or Other | 401 | 122 | 0.58 | 0.29, 1.12 | 0.106 |
| **Policy environment layer** |  |  |  |  |  |
| Medical Insurance |  |  |  |  |  |
| Urban and rural residents' Medical insurance | 1670 | 538 | Reference | - | - |
| Urban Employees' Medical Insurance | 515 | 113 | 0.41 | 0.22, 0.75 | 0.004 |
| Out-of-pocket or Other | 39 | 18 | 0.34 | 0.09, 1.35 | 0.126 |
| hospital.sd (Intercept) | 2,224 | 669 | 0.19 |  |  |
| ^1^OR = Odds Ratio, ^1^CI = Confidence Interval | | | | | |

Table S4. Goodness-of-fit Indices for the Multilevel Logistic Regression Model

| Fit Index | Value |
| --- | --- |
| AIC | 958.89 |
| BIC | 1,084.45 |
| Conditional R² | 0.766 |
| Marginal R² | 0.764 |
| ICC | 0.011 (1.14%) |
| Variance of Random Intercept | 0.038 |
| Standard Deviation of Random Intercept | 0.195 |
| Log-Likelihood | -457 |
| Number of Groups (Centers) | 10 |
| Number of Observations | 2,224 |
| AIC=Akaike Information Criterion;BIC=Bayesian Information Criterion;ICC=Intra-class Correlation Coefficient | |


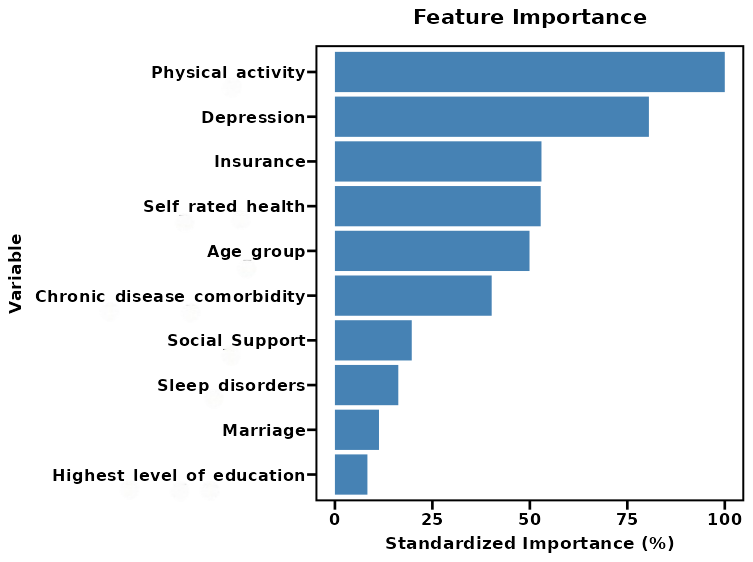


Figure S1. Standardized feature importance plot for the BPNN model.

Table S5. Detailed Performance Metrics and Confusion Matrices for the BPNN Model

| Dataset | Accuracy | Sensitivity | Specificity | PPV | NPV | Confusion Matrix (TN, FP, FN, TP) |
| --- | --- | --- | --- | --- | --- | --- |
| Training | 0.947 | 0.970 | 0.891 | 0.956 | 0.923 | [1099, 50, 34, 409] |
| Test | 0.910 | 0.953 | 0.824 | 0.916 | 0.896 | [402, 37, 20, 173] |
| Abbreviations: TN, True Negative; FP, False Positive; FN, False Negative; TP, True Positive; PPV, Positive Predictive Value; NPV, Negative Predictive Value. | | | | | | |


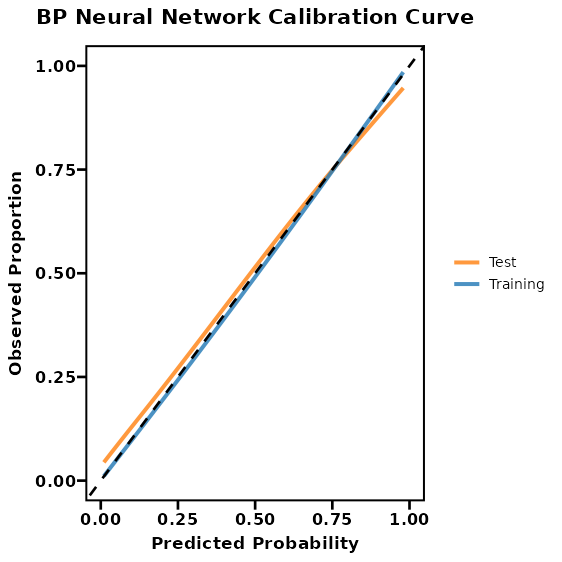


Figure S2. Calibration Plots for the BPNN Model
